# Potential antidiabetic activity of benzimidazole derivative albendazole and lansoprazole drugs in different doses in experimental type 2 diabetic rats

**DOI:** 10.3906/sag-2004-38

**Published:** 2021-06-28

**Authors:** Burak DİK, Devran COŞKUN, Emre BAHÇİVAN, Kamil ÜNEY

**Affiliations:** 1 Department of Pharmacology and Toxicology, Veterinary Faculty, SelÇuk University, Konya Turkey; 2 Department of Pharmacology and Toxicology, Veterinary Faculty, Siirt University, Siirt Turkey; 3 Department of Pharmacology and Toxicology, Veterinary Faculty, Kafkas University, Kars Turkey

**Keywords:** Benzimidazole, HOMA-β, diabetes, antiabetic, rat

## Abstract

**Background/aim:**

The aim of this study is to determine the effects of different concentrations of albendazole and lansoprazole, which were benzimidazole derivatives, on endocrinologic and biochemical parameters in experimental type 2 diabetic (T2D) rats.

**Materials and methods:**

In this study, 46 male Wistar Albino rats were used. Animals were divided as healthy control (0.1 mL/rat/day saline, s.c, n = 6), diabetes control (0.1 mL/rat/day saline, s.c, n = 8), diabetes+low-dose albendazole (5 mg/kg, oral, n = 8), diabetes+high-dose albendazole (10 mg/kg, oral n = 8), diabetes+low-dose lansoprazole (15 mg/kg, subcutaneous, n = 8), and diabetes+high-dose lansoprazole (30 mg/kg, subcutaneous, n = 8). All groups were treated for 8 weeks. The blood samples were analyzed by autoanalyzer and ELISA kits for biochemical and endocrinological parameters, respectively.

**Results:**

Glucose, HbA1c, triglyceride, low density cholesterol (LDL), leptin, and Homeostatic Model Assessment for insulin resistance (HOMA-IR) levels increased and insulin and HOMA-β levels decreased in the diabetic rats compared to the healthy control group. The glucose, HbA1c, and triglyceride levels were partially decreased; however, insulin and HOMA-β levels were increased by low-dose albendazole therapy. The high dose of lansoprazole treatment increased insulin level.

**Conclusion:**

The lansoprazole and albendazole treatments can be a potential drug or combined with antidiabetic drugs in T2D treatment by *Adenosine 5* ′ *-monophosphate* activated protein kinase (AMPK), peroxisome proliferator-activated receptor (PPAR), *incretin-like effect* and other antidiabetic mechanisms. It may be beneficial to create an effective treatment strategy by developing more specific substances with benzimidazole scaffold.

## 1.Introduction

Type 2 diabetes is defined as a chronic inflammatory metabolic disease that is characterized with the impaired insulin effect and high blood glucose level [1]. The disease has degenerative effects on many tissues and a high incidence worldwide. Although many drugs are used for the treatment of this disease, more effective treatment strategies have yet to be found [2,3]. 

Adenosine 5′-monophosphate activated protein kinase (AMPK) is considered to be an important potential therapeutic target for the treatment of type 2 diabetes (T2D). AMPK is defined as serine/threonine protein kinase complex. The kinase consists catalytic α (α1, α2), regulatory β (β1, β2), and regulatory γ (γ1, γ2, γ3) subunits and express in each tissue [3]. Although the isoforms have been reported to have different effects and tissue-specific, the effects of isoforms have not been fully cleared. However, α1 isoform predominates in the liver and adipose tissue; whereas, α2 predominates in the brain, heart, and skeletal muscles [4]. The AMPK acts as a sensor that determines the AMP/ATP or ADP/ATP ratio. The AMPK is activated by phosphorylation of Thr-172 residue in the α subunit and increasing AMP/ATP ratio in the cell [3-6]. The activated AMPK provides the phosphorylation of key metabolic proteins that inhibits anabolic activities and increase catabolic activities [3]. The AMPK activation suppresses genes mediating gluconeogenesis and lipogenesis in the liver [7], and the activation of AMPK has been reported to increase insulin secretion from pancreatic β cells [3,8]. On the contrary, the excessive increased insulin, leptin and diacylglycerol levels and hyperglycemia, hyperlipidemia inhibit the AMPK activation [6]. Therefore, the AMPK activation increases insulin sensitivity, stimulates glycogen synthesis while inhibits glycolysis. Thus, it is an important therapeutic target for glucose hemostasis in the treatment of T2D patients [3,4,6,8]. 

Metformin, one of the most important antidiabetics, provides indirect activation of AMPK [3,7]. In addition, thiazolidinedione, which is also an antidiabetic drug and nondrug ginsenosids, berberine, quercetin, and resveratrol indirectly activate the AMPK [3,9]. Recently, potential antidiabetic agents (AICAR, A769662, PT1, C24, MK-8722, PF-06409577, ex229, PF-06409577 and benzimidazole compound 991) that enable the AMPK activation of skeletal muscles and liver have been tested for T2D treatment [3]. The ex229, newly developed benzimidazole derivative and the AMPK activator, stimulates glucose entry into skeletal muscles and increases oxidation of fatty acids in skeletal muscles. Moreover, the antidiabetic effects of ex229 are variable as depending on the dose [10]. Metformin has also been shown to reduce insulin resistance, hyperglycemia, and it increases dose-dependent glucose entry into the cell by the dose-dependent effect and the indirect AMPK activation [11]. Metformin contributes to glucagon-like peptide (GLP)-1 secretion by AMPK activation and potentiates reducing blood glucose [12]. Metformin has inhibited the lipogenesis and gluconeogenesis, and it regulates the glucose transport, glycolysis, glycogenesis and fatty acid oxidation by activating AMPK α1 and α2 transcription in fish fed a high with energy diet [13]. Metformin and AICAR administration have restricted the hepatic production of glucose by the phosphorylation of Thr172 region of the AMPK [14]. Antidiabetic drugs troglitazone and pioglitazone have increased glucose entry into muscle cells and fatty acid oxidation in muscle cells by the AMPK activation. Also, troglitazone has activated the AMPK signal in liver and adipose tissue and increases AMP/ATP ratio [15].

Albendazole, a benzimidazole derivative, is generally used as an anthelmintic in most mammals. It is defined as an enantiomeric drug. Albendazole is converted to the sulfoxide structure in the liver and distributes widely to tissues [16]. In addition, albendazole has been reported to provide the AMPK activation in vitro and in vivo [17]. Lansoprazole, another benzimidazole derivative, has been used for the treatment of diseases such as ulcers and esophagitis for about 27 years [18]. Although lansoprazole is not reported the direct effect on the AMPK, it regenerates and sensitizes beta cells, induces peroxisome proliferator-activated receptor-γ (PPARγ) through incretin and the regulation of lipid metabolism, and increases the insulin synthesis and adiponectin/leptin ratio [19-22]. In addition, its high doses decrease glucose, triglyceride levels, and stimulate beta cells by increasing incretin secretion, providing β cell regeneration and neogenesis, delaying gastric emptying [19-23]. 

In recent years, alternative treatment strategies have been tried in the treatment of T2D. Benzimidazole-derived substances have antidiabetic potentials by affecting different mechanism, especially the AMPK pathway. The aim of this study was to determine the efficacy of benzimidazole-derived albendazole (5 mg/kg and 10 mg/kg) and lansoprazole (15 mg/kg and 30 mg/kg) on different endocrinological and biochemical parameters in experimental T2D rats.

## 2.Materials and methods

### 2.1. Animals

The study was carried out on 46 healthy male Wistar Albino rats aged 8-12 weeks. The fasting blood glucose and basal lipid profiles of rats were determined before the study. Feed and water requirements were met as ad libitum. The composition of the ration given to the healthy control group is as follows: dry matter: 89%, crude protein: 21%, cellulose: <5%, ash <10%, Ca: 1%–2%, P: 0.5%–1%, NaCl: 0.5%, ME: 2850 kcal/kg. The animals were housed in Selçuk University Chair of Experimental Medicine Research and Application Center (SUCEMRAC), Konya, Turkey. Research protocol was reviewed and approved by the Ethic Committee of SUCEMRAC (Approved number: 2017-41).

### 2.2. Induction of Type 2 diabetes

Experimental Type 2 diabetes (T2D) model was induced according to the method proposed by Srinivasan et al [24]. In this method, rats were fed by high fat diet (HFD, containing 58% fat, 25% protein and 17% carbohydrate, as a percentage of total kcal) ad libitum for 2 weeks. HFD composition included 37% animal fat as tallow, 30.5% corn, 3% vegetable oil, 20% casein, 4.5% soy pulp, 1.7% dicalcium phosphate, 0.2% dl-methionine, 1.6% limestone, 0.5% salt, and a vitamin-mineral blend 1%. The streptozotocin (STZ, 35 mg/kg, s.c.) were administered to rats after the 2 weeks. Before injection, streptozotocin (STZ, ≥98%, analytical purity, Sigma-Aldrich Corp., St. Louis, MO, USA) was dissolved in citrate buffer (pH 4.5, 20 mg/mL). After the injection, 5 % dextrose was supplemented to prevent the animal from fatal hypoglycemia for 24 h. The rats with the nonfasting glucose level of ≥300 mg/dL were considered as diabetic on 5 days following STZ injection. The diabetic rats were fed by HFD ad libitum until the end of the study. 

### 2.3. Drug preparation

Albendazole was used commercially solution (Vermiprazole 10%, oral solution, Hipra, Spain) formulation. Lansoprazole (≥98%, analytical purity) was provided in powder form from Tokyo Chemical Industry (Product Number-L0233, Japan). Lansoprazole was dissolved in solution containing 0.9% sodium chloride and 0.05% sodium hydroxide in water as 30 mg/mL. After preparing the stock solutions of the drugs, they were diluted with 0.9% sodium chloride to the appropriate dosage amounts.

The doses of the drugs, which were chosen according to EMEA data, used in the current research were determined experimentally in the experimental research, and the standard doses were indicated in the drug package insert.

### 2.4. Experimental design

The rats were housed in standard polypropylene cages during the study. The light (12/12 h light/dark cycle), temperature (22 ± 2 ° C) and humidity (55 ± 5%) of the room were maintained under control. All the rats were grouped as follows.

The saline (0.1 mL/rat/day, s.c.) solution were administered to group 1 (Healthy control, n = 6) and group 2 (Diabetes control, n = 8) during the experiment. Group 3 [Diabetes+Low-Dose Albendazole (n: 8)] and group 4 [Diabetes+High Dose Albendazole (n: 8)] received albendazole at 5 mg and 10 mg/kg/day by oral for 8 weeks, respectively. Group 5 [Diabetes+Low-Dose Lansoprazole (n: 8)] and group 6 [Diabetes+High Dose Lansoprazole (n: 8)] received lansoprazole at 15 mg and 30 mg/kg/day by subcutaneously for 8 weeks, respectively.

The healthy control rats were fed with the commercially available rat normal pellet diet, and all of the diabetic rats were fed with HFD and all of the rats drank water ad libitum. The blood samples were collected by retro-orbital sinus at the end of week 8 under thiopental Na anesthesia (40 mg/kg, i.p.) and separated as serum. Then, the animals were euthanized by cervical dislocation. The whole blood samples for HbA1c were analyzed immediately while the serum samples were stored at -800 C until analysis.

### 2.5. Biochemical, endocrinological, and antioxidant capacity analysis

Biochemistry parameters (HDL: high density cholesterol, LDL: low density cholesterol, AST: aspartate aminotransferase, ALT: alanine aminotransferase, glucose, urea, creatinine, cholesterol, triglyceride) in serum samples and HbA1c (Hemoglobin A1c) parameter in whole blood samples were analyzed by the autoanalyser (BT-300 plus, Rome, Italy) and HPLC (Primus Primatech USA), respectively. Insulin (Ultra Sensitive Rat Insulin ELISA Kit, Catalog no: 90060, Crystal Chem, USA), adiponectin (Rat ADP / Acrp 30 ELISA Kit, Catalog no: E-EL-R0329, Elabscience Biotechnology Co. Ltd., China), resistin (Rat RTN ELISA Kit, Catalog no. E-EL-R0614, Elabscience Biotechnology Co. Ltd., China), leptin (Rat LEP ELISA Kit, Catalog no. E-EL-R0582, Elabscience Biotechnology Co. Ltd., China), and total antioxidant capacity marker (TAC) (Total Antioxidant Capacity Assay Kit, Catalog no: ab65329, Abcam Company, United Kingdom) were determined following to manufacturer’s protocol by the ELISA reader (Bio-Tek Instruments Inc., MWGt Lambda Scan 200).

Homeostatic Model Assessment for insulin resistance (HOMA-IR) and β-cell activity (HOMA-β) were calculated according to the following formula using with the determined glucose and insulin values determined in the study.

HOMA-IR: (Glucose × Insulin) / 405, 

HOMA-β: (360 × Insulin) / (Glucose − 63).

### 2.6. Statistical analysis

The data were analyzed on SPSS 25.0 (SPSS, Inc., Chicago, IL, USA) software, and all values were evaluated as median [interquartile range (IQR)]. Statistical significance between groups was tested using the Kruskal-Wallis and post hoc Dunn-Bonferoni test. The data was considered statistically significant at p < 0.05.

## 3. Results

The changes in various biochemical parameters after lansoprazole (15 and 30 mg / kg / day, SC) and albendazole (5 and 10 mg / kg / day, oral) treatment in experimental T2D are illustrated Table 1. The endocrinological data are presented in Table 2 and HOMA results are presented in Table 3. 

**Table 1:** Effect of lansoprazole (15 and 30 mg/kg, SC) and albendazole (5 and 10 mg/kg/day, oral) treatment on biochemical parameters in type 2 diabetic rats [median (IQR)].

Parameters	Healthycontrol	Diabetescontrol	Diabetes +Low-dose Lansoprazole	Diabetes +High-dose Lansoprazole	Diabetes +Low-dose Albendazole	Diabetes +High-dose Albendazole
Glucose (mg/dL)	129.5(122.5–158.8)	486.5 #(295.8–522.8)	467.0 #(430.5–469.5)	477.0 #(473.0–493.0)	221.5(187.3–287.5)	433.0 #(206.5–492.5)
HbA1c (%)	5.3(5.0–5.5)	12.1 #(7.3–12.7)	12.0 #(11.5–13.1)	12.0 #(11.4–12.7)	7.15(6.0–9.3)	10.5 #(8.2–11.1)
Triglycerides (mg/dL)	171.0(149.5–183.3)	435.5 #(387.5–557.0)	484.0(143.5–635.0)	457.0 #(271.0–536.5)	356.5(197.5–472.3)	465.5 #(432.5–681.5)
Cholesterol (mg/dL)	71.0(67.0–99.3)	139.0(72.3–334.8)	86.0(76.5–92.5)	77.0(72.5–227.5)	77.5(72,5–83.0)	108.0(87.0–187.5)
HDL (mg/dL)	50.0(47.8–74.3)	45.0(36.5–52.8)	34.0(26.0–57.5)	30.0(28.5–39.5) #	45.5(42.8–55.3)	38.0(31.5–58.0)
LDL (mg/dL)	7.69(6.0–9.0)	19.5 #(14.5–47.8)	21.0 #(15.5–32.0)	20.0 t(18.0–30.0)	11.0 *(10.3–12.8)	22.4 #(13.5–33.5)
AST (U/L)	136.5(106.8–149.0)	67.5 #(39.0–99.0)	102.0(91.0–124.5)	88.0 t(60.0–99.0)	81.0 #(70.3–95.8)	75.0 #(70.3–95.8)
ALT (U/L)	69.0(58.3–75.5)	31.0 #(25.8–46.8)	54.0 *(34.5–61.5)	48.0(34.5–61.5)	28.0 #(23.3–40.0)	37.0 #(28.0–50.0)
Urea (mg/dL)	45.0(42.8–53.5)	43.0(35.0–48.8)	41.0(36.5–76.5)	32.0 *(29.5–36.5)	38.5 #(30.5–45.0)	39.0 #(30.5–42.0)
Creatinine (mg/dL)	0.50(0.46–0.54)	0.55(0.49–0.60)	0.61 t, *(0.57–0.63)	0.59 #(0.57–0.61)	0.53(0.50–0.55)	0.54 #(0.50–0.62)
TAS (nmol)	1.05 (1.0–1.31)	1.05 (1.05–1.15)	1.08(1.02–1.23)	1.09(1.0–1.17)	1.18 (1.13–1.25)	1.08(1.05–1.11)

# denotes significant difference vs. healthy control group at p ˂ 0.05.* denotes significant difference vs. diabetes control group at p ˂ 0.05.HDL: High density cholesterol, LDL: Low density cholesterol, AST: Aspartate aminotransferase, ALT: Alanine aminotransferase, HbA1c (%): Hemoglobin A1c, TAS: Total antioxidant status.

**Table 2:** Effect of lansoprazole (15 and 30 mg/kg, SC) and albendazole (5 and 10 mg/kg/day, oral) treatment on endocrinological parameters in type 2 diabetic rats [median (IQR)].

Parameters	Healthycontrol	Diabetescontrol	Diabetes +Low-dose Lansoprazole	Diabetes +High- dose Lansoprazole	Diabetes +Low- dose Albendazole	Diabetes +High- dose Albendazole
Insulin (ng/mL)	1.30 (1.16–1.69)	0.93 # (0.85–0.98)	1.19 (1.06–1.29)	1.42 *(0.98–1.54)	1.41 *(1.34–1.65)	1.18 (1.05–1.27)
Adiponectin (pg/mL)	128.0(58.5–162.5)	53.0 (30.8–131.5)	7.0(0–118.5)	0 (0–56)	79.0(45.5–458)	175.0(28.3–342)
Resistin (ng/mL)	0.19(0.6–2.84)	4.10(1.74–5.94)	0(0–2.39)	0(0–8.30)	0.63(0.38–1.83)	0(0–0.82)
Leptin (ng/mL)	1.49(0.27–2.24)	38.2 #(6.63–56.9)	9.41 #(6.83–19.26)	7.05 t(3.65–20.8)	3.32 (2.60–7.20)	8.48 #(3.27–22.9)

# denotes significant difference vs. healthy control group at p ˂ 0.05.* denotes significant difference vs. diabetes control group at p ˂ 0.05.

**Table 3:** Effect of lansoprazole (15 and 30 mg/kg SC) and albendazole (5 and 10 mg/kg/day, oral) treatment on HOMA parameters in type 2 diabetic rats [median (IQR)].

Parameters	Healthycontrol	Diabetescontrol	Diabetes +Low-dose Lansoprazole	Diabetes +High- dose Lansoprazole	Diabetes +Low- dose Albendazole	Diabetes +High- dose Albendazole
HOMA-IR	11.19(10.4–12.7)	26.37 #(25.9–27.8)	32.27 #(29.2–35.8)	27.45 #(16.8–38.8)	19.52 #(16.3–37.7)	21.0 #(14.0–31.0)
HOMA–β	160.4(102.5–235.8)	19.4 #(16.1–21.4)	26.5 #(22.5–27.7)	36.0 #(28.0–138.2)	78.5*(28.0–138.2)	48.03 #(25.4–73.7)

# denotes significant difference vs. healthy control group at p ˂ 0.05.* denotes significant difference vs. diabetes control group at p ˂ 0.05.

The glucose, HbA1c, triglyceride, and LDL values in the diabetes group increased compared to the control group (p < 0.05). Also, leptin and HOMA-IR levels increased, while insulin and HOMA-β levels decreased in the diabetes control group. 

The low-dose albendazole treatment (LDAT) partially decreased glucose, HbA1c, triglyceride, and leptin levels. However, LDAT significantly decreased LDL level and increased insulin and HOMA-β levels compared to diabetes control group (p < 0.05). 

The low-dose lansoprazole treatment (LDLT) decreased partially triglyceride level and increased insulin level however the high-dose lansoprazole treatment (HDLT) significantly increased insulin level compared to diabetes control group (p < 0.05). Although there were statistically significant changes in AST, ALT, urea, and creatinine values, these values were within the reported ranges for rats.

The insulin levels in the LHLT and LDAT groups were similar to healthy control group level. Besides, leptin and HOMA-β levels in the LDAT group were similar to healthy control group level.

Although there were no statistically significant differences in the treatment groups compared to the diabetes control group for adiponectin and resistin, its level in LDAT and high-dose albendazole treatments (HDAT) groups were higher 209% and 161% than diabetes control group, respectively (p > 0.05). The resistin levels decreased significantly in LDAT and HDAT, LDLT groups (p < 0.05, Table 2). 

## 4. Discussion

Many studies have shown that AMPK activation is a key mechanism in antidiabetic treatment [12,25]. Some benzimidazoles have been reported to have antidiabetic effects by mainly activation of AMPK and PPAR, inhibition of α-amylase and α-glucosidase enzymes and other antidiabetic mechanisms.[10,26-28]. However, these effects on AMPK may vary depending on the dose, the ratio of binding to the AMPK, and activating different subunits of AMPK [5]. Therefore, the benzimidazoles can be considered as multitarget antidiabetic agents.

Furthermore, some benzimidazole molecules cause regeneration of pancreas β cell, and the regulation of adipose tissue [21,22,26,27]. It was emphasized that telmisartan and lansoprazole, benzimidazole derivatives, could be used as an antidiabetic in the treatment of T2D by activating PPAR [27]. Other benzimidazole derivatives compound 991 and lansoprazole have increased glucose transport into tissues by AMPK activation or other mechanism [22,29,30]. Metformin contributes to the anthelmentic effect of albendazole by providing AMPK activation in the larval period of *Echinococcus granulosus* [31]. 

In the present study, the LDAT may prevent gluconeogenesis and increase the effects of glucose transporters by providing the AMPK activation in liver and skeletal muscles. Also, the effects of albendazole on the inhibition of α-amylase and α-glucosidase may have contributed to its antidiabetic effect. In the literature, the antidiabetic effects of benzimidazoles have been reported to be dose-dependent, and the LDAT may have resulted partially decrease in glucose and HbA1c in the current study. In addition, the LDAT and LDLT can cause partially the reduction in triglyceride levels by suppressing lipogenic genes and AMPK activation. The LDAT may regulate lipoprotein lipase in muscles by activation of the PPAR pathway and LDAT significantly decreased LDL level and partially increased HDL level. However, the short duration of the current study and the feeding of HFD (strong diabetic and lipidemic effects) during the study may have masked the antidiabetic treatments. 

Biochemical parameters of the present study are observed to be within the reference range reported for rats [32,33] although there are statistical differences between groups in ALT, AST, creatinine, and urea values. In the present study, the biochemical changes show that there are no side effects of the drugs used for treatment in diabetic rats.

The LDLT and HDLT significantly increased insulin levels compared to diabetes group in the present study. This effect may occur as a result of β cell regeneration. Because the treatment provides regeneration of β cell through activation of PPARγ and AMPK [3,8,22]. HOMA-β, an indicator of B cell functionality, was significantly higher in the low-dose albendazole treatment group from the diabetes group. This shows that LDAT may provide regeneration of β cells through more than one antidiabetic pathway.

Adiponectin levels has negative correlation, while leptin and resistin levels has positive correlation with insulin resistance in diabetic individuals [34]. The increase of leptin level induces the formation of free oxygen radicals. This situation triggers hyperglycemia, adiponectin level and leads to insulin resistance [22]. In the present study, the positive effect of LDAT via PPAR and AMPK effects on insulin resistance may cause the decrease in leptin and resistin levels and the increase in adiponectin levels. However, insulin resistance may be inadequately treated by lansoprazole treatments, as the leptin level did not decrease enough in the current study. As lipid-lowering effects of higher doses of lansoprazole treatments have been reported [22,23], the albendazole treatments might prevented the increase of leptin level in the current study. This effect may be reduction of adipose tissue mass because it decreased the leptin synthesizes in adipose tissue [5,6]. Therefore, the percentage changes in adiponectin level could be caused by the regulator role of insulin in fat cells and the differences in the effects of treatment on organs [35,36]. The positive effects of albendazole on insulin and the short duration of treatment for diabetes may have caused partial changes in adiponectin level. Resistin is an important factor for insulin resistance; however, its expression reportedly changed in two different directions (increase / decrease) by treatments in different studies [35,36]. In the current study, the low-dose albendazole treatments may decrease the insulin resistance and hyperlipidemia through AMPK, PPARγ, and other pathways.

Metformin has been caused a nonsignificant change in HOMA-IR, although it has been activated the AMPK in the obese mice [37]. Also, berberine was reported to be antidiabetic effects by AMPK activation and decreased HOMA-IR value and insulin resistance [38]. However, the AMPK activation may produce different effects because the activation of different subunits of AMPK has effective at different ability and potential in different tissues [4,39]. Although all the treatments in the current study could not change HOMA-IR levels, these treatments may prevent gluconeogenesis in the liver and improve the pancreatic regeneration by AMPK activation. This situation can be confirmed by the increase in HOMA-β and insulin levels, in particularly albendazole treatments groups.

In conclusion, new studies are carried out to show that different benzimidazole derivatives may have antidiabetic effects through AMPK, PPARγ, α-glucosidase, and other pathways. Structural compatibility of benzimidazoles on antidiabetic mechanisms is especially important [28,40,41]. The AMPK and PPARγ activation have been considered as alternative important targets for the treatment of T2D and obesity in recent years. The detailed research with benzimidazole has not been conducted yet, because antidiabetic mechanisms of benzimidazoles are just under investigation. The differences between albendazole and lansoprazole may have been caused by the difference in the 3-dimensional structure of benzimidazoles that is important for AMPK-drug interaction, the dose of the drugs and activation of AMPK and PPARγ in different organs. 

Further studies including molecular and biochemical mechanisms, pharmacodynamics, molecular docking, and pharmacophore model is necessary to determine the antidiabetic effect of various benzimidazole (particularly albendazole) at different doses in T2D treatment. In addition, comparative studies between AMPK activator benzimidazoles and antidiabetic drugs such as metformin and thiazolidinediones will contribute to the effective antidiabetic treatment in future.
